# Incidence of drug-resistant pathogens in community-acquired pneumonia at a safety net hospital

**DOI:** 10.1128/spectrum.00792-24

**Published:** 2024-07-16

**Authors:** Helen Ding, Norman S. Mang, Jordan Loomis, Jessica K. Ortwine, Wenjing Wei, Ellen J. O’Connell, Nainesh J. Shah, Bonnie C. Prokesch

**Affiliations:** 1 Department of Pharmacy, Parkland Health, Dallas, Texas, USA; 2 Department of Emergency Medicine, University of Texas Southwestern Medical Center, Dallas, Texas, USA; 3 Department of Internal Medicine, University of Texas Southwestern Medical Center, Dallas, Texas, USA; Icahn School of Medicine at Mount Sinai, New York, New York, USA

**Keywords:** antibiotic stewardship, community-acquired pneumonia, multidrug-resistant organism, methicillin-resistant *Staphylococcus aureus*, *Pseudomonas aeruginosa*, incidence

## Abstract

The 2019 Infectious Diseases Society of America guideline for the management of community-acquired pneumonia (CAP) emphasizes the need for clinician to understand local epidemiological data to guide selection of appropriate treatment. Currently, the local distribution of causative pathogens and their associated resistance patterns in CAP is unknown. A retrospective observational study was performed of patients admitted to an 870-bed safety net hospital between March 2016 and March 2021 who received a diagnosis of CAP or healthcare-associated pneumonia within the first 48 hours of admission. The primary outcome was the incidence of CAP caused by methicillin-resistant *Staphylococcus aureus* (MRSA) or *Pseudomonas aeruginosa* (PsA) as determined by comparing the number of satisfactory sputum cultures or blood cultures with these drug-resistant organisms to the total number of reviewed patients. Secondary outcomes studied included risk factors associated with CAP caused by drug-resistant organisms, utilization of broad-spectrum antibiotics, appropriate antibiotic de-escalation within 72 hours, and treatment duration. In this 220-patient cohort, MRSA or PsA was isolated from three sputum cultures and no blood cultures. The local incidence of drug-resistant pathogens among the analyzed sample of CAP patients was 1.4% (*n* = 3/220). The overall incidence of CAP caused by MRSA or PsA among admitted patients is low at our safety-net county hospital. Future research is needed to identify local risk factors associated with the development of CAP caused by drug-resistant pathogens.

**IMPORTANCE:**

This study investigates the incidence of drug-resistant pathogens including methicillin-resistant *Staphylococcus aureus* and *Pseudomonas aeruginosa* among community-acquired pneumonia (CAP) patients at a safety net hospital. Understanding local bacteria resistance patterns when treating CAP is essential and supported by evidence-based guidelines. Our findings empower other clinicians to investigate resistance patterns at their own institutions and identify methods to improve antibiotic use. This has the potential to reduce the unnecessary use of broad-spectrum antibiotic agents and combat the development of antibiotic resistance.

## INTRODUCTION

Community-acquired pneumonia (CAP) is a frequently encountered infection of the lung parenchyma which is a leading cause of hospitalizations, plays a major role in reported mortality rates, and incurs significant healthcare costs (1, 2). Pathogens most commonly responsible for CAP include typical bacteria such as *Streptococcus pneumoniae* and *Haemophilus influenzae*, atypical bacteria species including *Mycoplasma pneumoniae* and *Chlamydia pneumoniae*, as well as various respiratory viruses (3). Methicillin-resistant *Staphylococcus aureus* (MRSA) and *Pseudomonas aeruginosa* (PsA) are less common etiologies of CAP compared to more routinely identified organisms. Despite the lower frequency of disease due to MRSA and PsA, utilization of broad-spectrum antibiotics targeting these organisms remains high in hospitalized patients with CAP and has promoted the emergence of drug-resistant pathogens (4, 5). Because successful management of CAP is highly dependent on the use of antibiotics, appropriate selection is the key to effectively treating the offending organism while limiting its contribution to bacterial resistance.

Previously, the 2005 joint Infectious Disease Society of America (IDSA) and American Thoracic Society (ATS) guideline for the management of adults with hospital-acquired pneumonia, ventilator-associated pneumonia, and healthcare-associated pneumonia (HCAP) proposed several criteria to identify patients with pneumonia presenting from the community who are more likely to harbor drug-resistant pathogens, such as MRSA or PsA. These risk factors included residence in a nursing home or long-term care facility, hospitalization for ≥48 hours in the last 90 days, receipt of home infusion therapies such as antibiotics or chemotherapy, home wound care, chronic dialysis, and having a family member with a known antibiotic-resistant pathogen. If a patient met the criteria for HCAP, the previously recommended empiric treatment consisted of one agent with MRSA activity in addition to two agents from different pharmacological classes with PsA activity (6).

In response to these guideline recommendations, the use of anti-infective agents in CAP with activity against MRSA and PsA surged (7). Despite this, few apparent differences in patient outcomes were noted (8–10). Ultimately, the definition of HCAP was found to be neither sensitive nor specific in identifying CAP caused by MRSA or PsA, and the classification of HCAP was dissolved (7). Other methods, such as the drug resistance in pneumonia score, have been developed to aid in detecting risk factors for CAP caused by a drug-resistant organism (11). However, these scoring systems have failed to demonstrate consistency in improving patient outcomes or reducing broad-spectrum antibiotic use (12–14).

Despite the lack of a universally validated risk stratification tool, current evidence suggests that prior respiratory isolation of MRSA or PsA within the previous 12 months plays an important role in the development of pneumonia due to a drug-resistant organism (4, 15). Additionally, recent hospitalization and exposure to parenteral antibiotics within the previous 90 days warrants consideration of broad-spectrum antibiotic use (16, 17). The most recent guideline for the diagnosis and treatment of adults with CAP, published in 2019 by IDSA/ATS, recognizes the need for clinician to understand local epidemiological data to guide the selection of appropriate treatment (18). Therefore, the objective of this study is to determine the incidence of CAP caused by MRSA or PsA at our institution.

## MATERIALS AND METHODS

### Participants and design

This study was a single-center, retrospective observational study of a sample of 220 patients with pneumonia admitted between March 2016 and March 2021 to an 870-bed safety net hospital servicing a large population of uninsured and underinsured patients in North Texas. Patients were included if they received a clinical diagnosis of CAP or HCAP within 48 hours of admission. Patients were excluded if they had a diagnosis of SARS-CoV-2 infection on the same admission, had a history of SARS-CoV-2 infection requiring intubation, were chronically ventilated, or had an existing tracheostomy or laryngectomy. In accordance with the current IDSA/ATS guideline, we analyzed the frequency of MRSA or PsA as a CAP pathogen relative to the number of all CAP patients, not just those for whom cultures were sent (18). Satisfactory sputum cultures and an initial set of blood cultures obtained within 48 hours of admission were analyzed for each patient. Exemption from informed consent was granted by the hospital’s institutional review board given the retrospective nature of this study.

### Definitions

Patients were initially identified by diagnosis at the time of discharge, and the clinical diagnosis of CAP or HCAP was subsequently confirmed by reviewing provider notes from electronic medical records. A random cohort of patients was selected to represent all patients admitted with CAP diagnoses during the time period of interest. A classification of severe pneumonia was given to patients who were admitted to the intensive care unit within 48 hours of admission. Satisfactory sputum cultures were those with samples deemed acceptable for culture per the protocol of the microbiology laboratory requiring the sample to have less than 10 epithelial cells present. MRSA nares screening tests were performed using the CHROMAGAR MRSA assay, which is a qualitative culture-based test for the detection of MRSA colonization. Recent hospitalization was defined as admission for a duration of ≥48 hours within 90 days prior to the current admission. Recent parenteral antibiotic use was defined as receipt of parenteral antibiotics administered for active treatment purposes within 90 days prior to the current admission. Broad-spectrum antibiotics were those recommended for use against CAP caused by MRSA or PsA. The total duration of therapy included oral antibiotics that patients received following discharge. The immunocompromised state was defined as the presence of at least one of the following: congenital immunodeficiency, neutropenia (absolute neutrophil count < 1,000 cells/mm^3^), current use of immunosuppressive medications, recent (within three months) or current chemotherapy, human immunodeficiency virus (HIV) with CD4 cell count < 200 cells/mm^3^, or solid organ or bone marrow transplantation. Expected CAP organisms included *S. pneumoniae*, *S. aureus, H. influenzae*, *Moraxella catarrhalis*, *Legionella* species, *C. pneumoniae*, and *M. pneumoniae*.

### Study endpoints

The primary endpoint was the incidence of MRSA or PsA among patients admitted with CAP. Secondary endpoints included the percentage of initial antibiotic regimens involving a broad-spectrum agent, the percentage of initial broad-spectrum regimens de-escalated within 72 hours, and the duration of CAP antibiotic treatment. An additional area of interest was risk factors associated with the development of CAP caused by a drug-resistant organism.

### Procedures

Demographic information, individual risk factors, empiric antibiotic selection, broad-spectrum antibiotic de-escalation information, duration of treatment, microbiology data from sputum and blood cultures, and MRSA nares screening results were obtained from each patient’s electronic medical record. Descriptive statistics were performed to summarize CAP patient characteristics and assess antibiotic utilization. The incidence of CAP caused by drug-resistant organisms was determined by comparing the number of satisfactory sputum cultures or blood cultures with MRSA or PsA isolates to the total number of CAP patients.

## RESULTS

A total of 220 CAP patients meeting all inclusion and no exclusion criteria were evaluated. CAP patient characteristics are described in Table 1. There were 35 satisfactory sputum cultures and 208 blood cultures analyzed. From the 35 satisfactory sputum cultures collected, none yielded MRSA while three yielded PsA, and of the 208 blood cultures collected, none yielded MRSA or PsA. The primary endpoint, the incidence of MRSA or PsA as a cause of CAP, was 1.4% (*n* = 3/220) when comparing the isolation of drug-resistant organisms to the total number of patients analyzed with a CAP diagnosis (Table 2). MRSA nares screening tests were completed in 22 patients, among which one was positive (Table 3).

**TABLE 1:** CAP patient characteristics

Characteristic	Total (*n* = 220)
Age—mean (SD)	
Years	61 (11.2)
Sex*—n* (%)	
Female	111 (50.5)
Male	109 (49.5)
Race*—n* (%)	
Black	155 (70.5)
White	65 (29.5)
Ethnicity—*n* (%)	
Hispanic	37 (16.8)
Non-Hispanic	183 (83.2)
Comorbid conditions—*n* (%)	
Any comorbid condition, as below	159 (72.3)
Bronchiectasis	3 (1.4)
Cirrhosis	20 (9.1)
Chronic kidney disease	49 (22.3)
Chronic obstructive pulmonary disease	39 (17.7)
HIV	32 (14.5)
Immunocompromised	30 (13.6)
Type 2 diabetes mellitus	81 (36.8)
Social history—*n* (%)	
Any social history, as below	95 (43.2)
Active tobacco use	82 (37.3)
Homelessness	19 (8.6)
Residence in congregate care facility or group home	6 (2.7)
Residence in a nursing home or long-term care facility	8 (3.6)
Healthcare-related factors—*n* (%)	
Any healthcare-related factor, as below	81 (36.8)
Chronic dialysis	15 (6.8)
Hospitalization (within 90 days prior to admission)	73 (33.2)
Previous isolation of MRSA or PsA from sputum culture, within 1 year prior to admission	1 (0.5)
Previous isolation of MRSA or PsA from any non-sputum culture, within 1 year prior to admission	0 (0)
Previous positive MRSA nares swab (within 1 year prior to admission)	1 (0.5)
Receipt of intravenous antibiotics (within 90 days prior to admission)	40 (18.2)
Co-infections during the same admission—*n* (%)	
Influenza	1 (0.5)
Respiratory syncytial virus	1 (0.5)

**TABLE 2:** Incidence of MRSA or PsA based on culture data

Total CAP patients	Type of culture obtained	Number of patients with cultures^*a*^ obtained, *n* (%)	Number of patients with expected CAP organism isolated from culture	Number of patients with likely contaminant isolated from culture	Number of patients with MRSA isolated from culture	Number of patients with PsA isolated from culture	Incidence of CAP caused by MRSA or PsA among total CAP patients, *n* (%)
220	Sputum	35 (15.9)	3	1	0	3	3 (1.4)
	Blood	208 (94.5)	6	28	0	0	0 (0)

^*a*^ Includes satisfactory sputum cultures and an initial set of blood cultures obtained within 48 hours of hospital admission for CAP.

**TABLE 3:** MRSA nares screening test data^*a*^

Total CAP patients	MRSA nares screenings completed, *n* (%)	Number of positive MRSA nares screenings, *n* (%)	Patients who received empiric anti-MRSA treatment, *n* (%)
220	22 (10)	1 (0.5)	69 (31.4)

^*a*^ Abbreviations: CAP, community-acquired pneumonia; MRSA, methicillin-resistant *S. aureus*.

Secondary endpoint results are presented in Table 4. Broad-spectrum antibiotics with MRSA and/or PsA coverage were prescribed as initial CAP treatment in 32.3% (*n* = 21/220) of patients with 28.2% (*n* = 62/220) receiving antibiotics to cover both MRSA and PsA. Among patients receiving either, 40.8% (*n* = 29/71) had their broad-spectrum antibiotics discontinued within 72 hours. The most utilized initial antibiotic regimen was ceftriaxone and azithromycin which was prescribed in 47.7% (*n* = 105/220) of patients. The median duration of CAP treatment was 7 days (interquartile range: 2–4 days).

**TABLE 4:** Additional CAP patient characteristics and secondary outcome data^*a*^

Characteristic	Total (*n* = 220)
CAP severity—*n* (%)	
Non-severe	212 (96.4)
Severe	8 (3.6)
Patients initially receiving broad-spectrum therapy—*n* (%)	71 (32.3)
De-escalation within 72 hours from initiation of broad-spectrum initial therapy—n (% of patients initially receiving broad-spectrum therapy)	
Yes	29 (40.8)
No	42 (59.2)
Duration of antibiotic therapy—days	
Duration of IV therapy—median (IQR)	4 (2–4)
Duration of PO therapy—median (IQR)	4 (2–4)
Total duration of therapy—median (IQR)	7 (6–9)
Initial antibiotic therapy—*n* (%)	
Ceftriaxone	1 (0.5)
Ceftriaxone + azithromycin	105 (47.7)
Ceftriaxone + doxycycline	16 (7.3)
Ceftriaxone + moxifloxacin	2 (0.9)
Doxycycline	1 (0.5)
Moxifloxacin	24 (10.9)
Piperacillin/tazobactam	1 (0.5)
Piperacillin/tazobactam + moxifloxacin	1 (0.5)
Vancomycin + azithromycin	1 (0.5)
Vancomycin + ceftriaxone + azithromycin	5 (2.3)
Vancomycin + ceftriaxone + moxifloxacin	1 (0.5)
Vancomycin + aztreonam + azithromycin	1 (0.5)
Vancomycin + aztreonam + moxifloxacin	1 (0.5)
Vancomycin + cefepime	4 (1.8)
Vancomycin + cefepime + azithromycin	1 (0.5)
Vancomycin + cefepime + moxifloxacin	1 (0.5)
Vancomycin + meropenem	1 (0.5)
Vancomycin + piperacillin/tazobactam	22 (10.0)
Vancomycin + piperacillin/tazobactam + azithromycin	9 (4.1)
Vancomycin + piperacillin/tazobactam + ciprofloxacin	8 (3.6)
Vancomycin + piperacillin/tazobactam + ciprofloxacin + doxycycline	1 (0.5)
Vancomycin + piperacillin/tazobactam + doxycycline	6 (2.7)
Vancomycin + piperacillin/tazobactam + moxifloxacin	7 (3.2)

^*a*^ Abbreviations: IQR, interquartile range; PO, by mouth.

Due to the small number of patients with CAP caused by drug-resistant organisms, risk factors were not assessed for significance. However, the characteristics of the three patients with positive drug-resistant organism culture results are described in Table 5. Of the three patients involved, two had been hospitalized and received IV antibiotics within the previous 90 days. Of note, one patient had a positive MRSA nares screen but did not receive an MRSA-active agent as a component of empiric therapy. No modifications were made with regard to the antimicrobial spectrum based on the screening result due to rapid clinical improvement on the selected empiric regimen.

**TABLE 5:** Characteristics of CAP patients with drug-resistant organisms present in cultures obtained within 48 hours of admission

	Patient 1	Patient 2	Patient 3
Age	73	62	73
Sex	Female	Male	Female
Race	Black	Black	White
Ethnicity	Non-Hispanic	Non-Hispanic	Hispanic
Chronic obstructive pulmonary disease	−	+	−
Bronchiectasis	−	+	−
Cirrhosis	−	−	−
Type 2 diabetes mellitus	−	−	+
Chronic kidney disease	−	−	+
HIV	−	−	−
Immunocompromised	+	−	−
Active tobacco use	−	−	−
Nursing home or long-term care residence	−	−	−
Homelessness	−	−	−
Residence in a congregate care facility or group home	−	−	−
Prior hospitalization within the last 90 days	+	−	+
Previous isolation of MRSA or PsA from any culture (sputum or non-sputum), within 1 year prior to admission	−	−	−
Prior IV antibiotics within the last 90 days	+	−	+
Co-infections on same admission (influenza or respiratory syncytial virus)	−	−	−
Pneumonia severity	Non-severe	Non-severe	Non-severe
Initial antibiotic regimen	Vancomycin + piperacillin/tazobactam	Vancomycin + piperacillin/tazobactam + moxifloxacin	Moxifloxacin
Results from sputum culture obtained within 48 hours of admission	PsA	PsA	PsA
Results from blood culture obtained within 48 hours of admission	No growth	No growth	No growth
MRSA nares screening test result	Negative	Not obtained	Not obtained

## DISCUSSION

The primary purpose of this study was to identify the local incidence of drug-resistant organisms among CAP patients. The use of the HCAP classification is no longer supported by the current guideline, in part due to the varying frequency of drug-resistant organisms observed in CAP patients. In 2014, a systematic review and meta-analysis of 24 studies by Chalmers et al. found that MRSA prevalence varied from 0.7% to 30% in HCAP and 0% to 12% in CAP. PsA prevalence varied from 0.7% to 23% in HCAP and 0% to 8% in CAP (7). Jones et al. examined 38,473 CAP hospitalizations across 128 Veterans Affairs hospitals and found MRSA and PsA detection rates of 0.5% to 3.6% and 0.6% to 3.7%, respectively (19). Covington and Rufe observed a 3.6% prevalence of drug-resistant organisms among 527 CAP patients with culture results within 48 hours of admission to a community hospital in the United States (20). A larger cohort study of CAP patients in Barcelona, Spain by Cilloniz et al. found that MRSA was responsible for 2% of microbiologically confirmed patients (21).

Our study identified a 1.4% local incidence rate of PsA and a 0% incidence rate of MRSA as the causative organism for CAP based on satisfactory sputum and blood culture data from 220 patients admitted and diagnosed with pneumonia. While our findings are consistent with those of other studies, it was important to identify local resistance patterns given the variability in the prevalence of drug-resistant organisms in different populations and geographies. Notably, the frequency of broad-spectrum antibiotic use was unduly high relative to the low incidence of drug-resistant organisms in CAP patients at our institution. The use of agents with activity against MRSA and/or PsA as initial therapy was observed in nearly one-third of total patients with timely de-escalation occurring in less than half. We suggest closely following IDSA guidance to determine the need to add anti-MRSA or anti-pseudomonal coverage to promote antibiotic stewardship efforts and reduce unnecessary use of broad-spectrum antibiotics. In this study population, MRSA nares screening tests were performed in only 10% of CAP patients. While a zero incidence of CAP caused by MRSA in our sample suggests that broad-spectrum coverage is not needed for most patients, increasing the utilization of MRSA nares screening could be quite beneficial. MRSA nares swabs have a rapid turnaround time with results often available within 24 hours and are associated with high negative predictive values of 96% to 99%, thus allowing for timely antibiotic de-escalation in patients receiving empiric broad-spectrum treatment (22, 23).

Furthermore, despite the low number of patients meeting our definition of severe CAP (*n* = 8/220, 3.6%), blood cultures were obtained in 94.5% (*n* = 208) of total patients, with approximately one in eight blood cultures yielding an isolate considered to be a likely contaminant. Our data suggest that blood cultures should not be obtained among patients with non-severe CAP without another indication. Such a strategy will allow for potential reductions in cost, length of hospital stay, and duration of unnecessary broad-spectrum antibiotic use that may be associated with contaminated blood cultures.

Analysis of MRSA and PsA incidence was completed in accordance with IDSA recommendations. The 2019 IDSA CAP guideline highlights the need for local validation of MRSA and PsA prevalence in patients with CAP and identification of risk factors at a local level, citing the absence of high-quality outcome data, a very low incidence of MRSA or PsA at most centers, and significant increased use of anti-MRSA and anti-pseudomonal antibiotics for CAP treatment. Our study is the first at our institution to investigate the local incidence of drug-resistant organisms in CAP and provides valuable information about local epidemiology.

The median total duration of antibiotic therapy, including both IV and PO formulations, was observed to be 7 days. However, it is important to recognize that the current duration of therapy for CAP recommended by IDSA is as short as 5 days in clinically stable patients who are improving, including those with severe disease without infectious complications. This discrepancy indicates an opportunity to further improve antibiotic stewardship practices at our institution and encourage the adoption of evidence-based guidelines to minimize unnecessary antibiotic use.

Our study has several important limitations that should be acknowledged. First, our study design relied on discharge diagnoses to identify cases for inclusion and may have underestimated the true incidence of CAP. Descriptive statistics were used to describe CAP patient characteristics including demographics and potential risk factors for drug-resistant organisms. Few sputum specimens were satisfactory for culture, and acceptable samples were successfully obtained in only 15.9% of included patients (n = 35/220). Of note, there is no hospital-wide protocol requiring sputum cultures in all patients diagnosed with pneumonia at our institution. The total number of sputum specimens submitted for culture was not collected. Therefore, we were unable to draw conclusions about the utility and yield of sputum cultures in this patient population. Additionally, no conclusions could be drawn about specific risk factors for MRSA or PsA due to the small number of positive cultures. In addition, institutional criteria and initiation of a protocol for mandatory MRSA screening for all patients started on vancomycin for a respiratory indication were implemented in March 2019, which was in the middle of the study period. Prior to March 2019, there was not an institutional protocol for MRSA screening, and the test was rarely ordered. Lastly, the results of this study may not be generalized to other institutions or geographic areas.

Further research at our institution involving a larger sample size would add to our results and assist in identifying locally validated risk factors for CAP caused by MRSA or PsA, which will allow for further optimization of antibiotic treatment in this patient population.

### Conclusion

Based on microbiological data, the incidence of MRSA or PsA as a cause of CAP at our institution was found to be very low. This study offers valuable insight into local antibiotic resistance patterns and CAP treatment selections among our patient population and is consistent with patterns seen in other published studies. Furthermore, a high number of blood cultures were obtained in patients with non-severe CAP without additional indications, among which several contained organisms likely associated with contamination. Additionally, based on current prescribing practices, increased utilization of MRSA nares screening could be an effective tool in promoting more rapid de-escalation in patients initiated on empiric broad-spectrum antibiotics. The results of this study in conjunction with the current IDSA CAP guideline and publications in other patient cohorts with similar findings could be utilized to reduce unnecessary use of broad-spectrum antibiotics. In addition, this study highlights the importance of looking at local resistance patterns when determining empiric antibiotic therapy.
